# Occurrence and Profiles of the Artificial Endocrine Disruptor Bisphenol A and Natural Endocrine Disruptor Phytoestrogens in Urine from Children in China

**DOI:** 10.3390/ijerph121214964

**Published:** 2015-11-30

**Authors:** Mingyue Zhang, Zhenghua Duan, Yinghong Wu, Zhen Liu, Ke Li, Lei Wang

**Affiliations:** 1 Tianjin Centers for Disease Control and Prevention, Tianjin 300171, China; cdczhangmy@126.com (M.Z.); wuyinghongnk@126.com (Y.W.); 2 Ministry of Education Key Laboratory of Pollution Processes and Environmental Criteria/Tianjin Key Laboratory of Environmental Remediation and Pollution Control, Nankai University, Tianjin 300071, China; duanzhenghua@mail.nankai.edu.cn (Z.D.); m15822267505@163.com (Z.L.); like1030@mail.nankai.edu.cn (K.L.); 3 School of Environmental Science and Safety Engineering, Tianjin University of Technology, Tianjin 300384, China

**Keywords:** bisphenol A, phytoestrogens, biomonitoring, children, China

## Abstract

*Background*: Exposure to artificial or natural endocrine disruptors, such as bisphenol A (BPA) and phytoestrogens has been demonstrated to have health effects, especially in children. Biomonitoring of BPA and phytoestrogens in human urine can be used to assess the intake levels of these compounds. *Methods*: In this study, BPA and phytoestrogens in urine specimens (*n* = 256) collected from children in China were measured by liquid chromatography (LC)-tandem mass spectrometry (MS/MS). *Results*: BPA was detected in most specimens, with a geometric mean concentration of 1.58 ng/mL. For the first time, levels of urinary phytoestrogens in Chinese children were reported. Daidzein and enterolactone are the typical isoflavones and lignans compounds in urine, respectively. *Conclusions*: Relatively high levels of urinary BPA indicate an increasing risk of BPA exposure to Chinese children. Urinary concentrations of daidzein in Chinese children are higher when compared with those reported in the U.S. children, while concentrations of urinary enterolactone and enterodiols are significantly lower. This suggests a significant difference in phytoestrogen intake between the children from China and from the U.S.

## 1. Introduction

Exposure of exogenous chemicals can affect human health, which has resulted in an urgent need for human biomonitoring. For example, the Fourth National Report on Human Exposure to Environmental Chemicals, a national biomonitoring program in the U.S., mentions more than two hundred detected chemicals [1].

As an artificial chemical, 2,2-bis[4-hydroxyphenyl] propane (bisphenol A, BPA) is commonly used in the manufacturing of polycarbonate plastics and epoxy resins. BPA is of concern because it has been identified as an endocrine disrupting chemical (EDC) [2], implicated in reproductive and developmental anomalies in laboratory animals [3,4], and linked to a variety of adverse human health outcomes [5,6]. Biomonitoring studies have indicated a wide occurrence of detectable urinary BPA levels in humans from several countries [1,7,8,9,10,11,12,13,14].

Phytoestrogens are naturally occurring polycyclic phenols found in certain plants. Although numerous epidemiological and clinical studies have tried to evaluate the relationship between phytoestrogen consumption and human disease outcomes, the therapeutic potential of these compounds remains unclear [15]. On the contrary, these chemicals may have weak estrogenic effects when they are ingested and metabolized. The endocrine disrupting properties of phytoestrogens have been proposed in animals and in some epidemiological studies [15,16,17]. Isoflavones and lignans are two important groups of phytoestrogens. Two typical lignin urinary metabolites, *i.e.*, enterodiol and enterolactone, and four typical isoflavone urinary metabolites, *i.e.*, daidzein, genistein, equol, and *O*-desmethyl-angolensin (*O-*DMA), have been included in the U.S. national biomonitoring program [1]. However, knowledge about the occurrence of urinary phytoestrogens in the Chinese population is still insufficient, especially in Chinese children. Urine specimens are a common matrix for the biomonitoring of human exposure to chemicals. 

In the current study, 256 urine samples from children in Tianjin, China were collected, and the concentrations of BPA and six phytoestrogens compounds were determined using liquid chromatography (LC)-tandem mass spectrometry (MS/MS). Urinary concentrations of BPA and phytoestrogens are highly correlated with dietary intakes, and hence are valuable in assessing human exposure to these compounds.

## 2. Materials and Methods

### 2.1. Sample Collection

Tianjin is the fourth largest city in China, and also the industrial center and largest coastal city in Northern China. Urine specimens in this study come from a health and nutrition examination survey biomonitoring program of Tianjin Municipal Health Bureau. Probability proportion to size sampling was applied to collect urine specimens. In detail, during April and May 2014, 24 primary schools were randomly selected from the total of 956 schools in Tianjin. From each school, 11 students aged 8–10 years, were randomly invited to participate in urine specimen collection, after obtaining the consent of the children and their parents. All urine samples were collected in polypropylene (PP) tubes and shipped in dry ice to the laboratory within 6 h. In the laboratory, samples were stored at −80 °C before analysis. After excluding the losses during transportation and storage, urine samples (*n* = 256) from 132 boys and 124 girls, were measured for BPA and six phytoestrogens in the laboratory, with approval from the Ethics Committee of Nankai University.

### 2.2. Chemicals

Analytical standards of BPA (≥97%), enterolactone (95%), enterodiol (95%), daidzein (98%), equol (99%), genistein (98%), creatinine (99%) and *β*-glucuronidase (145700 units/mL *β*-glucuronidase and 887 units/mL sulfatase) were purchased from Sigma-Aldrich (St. Louis, MO, USA). *O*-DMA (98%) was purchased from Plantech (Reading, UK). Stable isotope-labeled standards, ^13^C_12_-BPA, D_3_-daidzein and D_4_-genistein were purchased from Cambridge Isotope Laboratories (Andover, MA, USA). D_3_-creatinine (99%) was purchased from CDN Isotopes (Pointe-Claire, QC, Canada).

### 2.3. Sample Pretreatment

To determine both the free and conjugated forms of the target analytes, 500 µL urine samples were transferred into a 15-mL PP tube, and 300 µL of 1.0 M ammonium acetate containing 22 units of *β*-glucuronidase, as well as ^13^C_12_-BPA, D_3_-daidzein and D_4_-genistein (5 ng of each), was added. After enzymatic deconjugation at 37 °C overnight, the digested samples were extracted by liquid-liquid extraction (LLE) and analyzed by LC-MS/MS. In brief, a digested sample was extracted twice by 4 + 4 mL of ethyl acetate, which was conducted with 60 min shaking each time. After centrifugation (5000× *g*, 5 min), the supernatants of the double LLE were combined and 1 mL of Milli-Q water was added to wash the extract. After that, extracts were concentrated to near-dryness under a gentle nitrogen stream, and redissolved in 0.5 mL of methanol for LC-MS/MS analysis. 

### 2.4. Chemical Analysis

Analysis of BPA and phytoestrogens was accomplished by an Acquity Ultra Performance LC system (Waters, Milford, MA, USA) interfaced with a Waters TQD tandem quadrupole mass spectrometer operated in the electrospray negative ion mode (ESI^−^-MS/MS). Ten microliters of the sample were injected onto an analytical column (Acquity UPLC BEH C_18_, 1.7 µm, 100 × 2.1 mm). The mobile phase comprised methanol (A) and Milli-Q water (B). A gradient elution was started from 35% (A), increased to 90% (A) at 0–2 min, to 96% (A) at 2–2.5 min, to 97% (A) at 2.5–5 min, to 100% (A) at 5–5.1 min and held for 0.9 min, then decrease to 35% (A) at 6–8 min and held 2 min before next injection. The flow rate was 300 mL/min. The MS/MS was operated in the multiple reaction monitoring (MRM). The MRM transitions monitored for BPA and phytoestrogens were similar to those mentioned in [18,19]. An isotope dilution method was used in analyte quantification. D_3_-daidzein was used for the quantification of enterolactone, enterodiol, equol, and *O*-DMA, given that no commercial iso-standards were available for these phytoestrogens. The recoveries of all target compounds spiked in matrices ranged from 81.3% to 113.7% (corrected by the recoveries of the internal standards). Creatinine was also detected by LC-MS/MS, according to the method described in our previous study [20].

### 2.5. Quality Assurance and Quality Control

For each batch of 20 samples, a procedural blank, a spiked blank, and a pair of matrix-spiked samples were analyzed. Procedural blanks were prepared by substitution of 0.5 mL of Milli-Q water for urine, followed by passage through the entire analytical procedure, indicating no target analytes detected in procedural blanks. The relative standard deviation (RSD) of replicate analysis of samples was <10%. The limit of quantification (LOQ) was 0.05 (for BPA), 2.50 (for enterolactone), 0.25 (for enterodiol), 9.00 (for daidzein), 5.00 (for equol), 0.25 (for *O*-DMA), and 1.5 ng/mL (for genistein), respectively, which was determined based on the lowest acceptable concentration in a calibration standard and a nominal sample volume of 0.5 mL. As a check for instrumental drift in response factors, a midpoint calibration standard was injected after every 10 samples; and a pure solvent (methanol) was injected as a check for cross-over from sample to sample. Instrumental calibration was verified by the injection of a 10-point calibration standard, and the regression coefficient (*r*) of calibration curves was ≥0.99.

### 2.6. Data Analysis

Statistical analyses were performed with SigmaPlot (Systat Software Inc., San Jose, CA, USA). For data analysis, concentrations below the LOQ were assigned a value equal to $LOQ/\sqrt{2}$ in the calculation of geometric mean (GM). Correlations between analytes were examined by Spearman’ correlation analysis. One-way analysis of variance was used to assess the differences between groups. A value of *p* < 0.05 was considered significant.

## 3. Results

Of the 256 urine specimens analyzed, BPA was found in 254 (99.2%) of the specimens (Table 1), indicating a widespread human exposure to BPA in the studied child population. The GM concentration of BPA was 1.55 ng/mL in children’s urine of, with GM urinary BPA concentrations of 1.32 ng/mL in girls and 1.73 ng/mL in boys, respectively. When adjusted by urinary creatinine, GM concentrations of urinary BPA in girls and boys were calculated to be 2.47 and 2.37 μg/g-creatinine, respectively (Table 1). 

**Table 1:** Occurrence of BPA and phytoestrogens in urine of children from Tianjin, China.

	Urinary Concentration (ng/mL)								Corrected by Urinary Creatinine (μg/g) ^c^						
	BPA	Enterolactone	Enterodiol	Daidzein	O-DMA	Genistein	Equol		BPA	Enterolactone	Enterodiol	Daidzein	*O*-DMA	Genistein	Equol
Children (*n* = 256)															
GM ^a^	1.58	162	21.4	142	4.87	21.7	10.8		2.52	258	34.1	226	7.76	34.6	17.2
AM ^b^	2.47	407	120	435	33.3	64.4	44.5		1.93	948	379	1350	71.6	204	182
Max	24.9	2910	4250	2720	346	419	336		74.3	258	34.1	226	7.76	34.6	29.6
DF%	99.2	100	98.4	93.4	71.9	98.4	53.1								
Girls (*n* = 124)															
GM	1.32	165	18.1	101	6.48	17.6	10.1		2.47	310	34.0	189	12.2	33.2	19.0
AM	1.99	366	48.8	379	37.0	58.7	38.7		2.11	1010	251	1320	107	242	70.6
Max	10.3	2910	595	2720	241	419	319		69.4	7090	4955	14,200	622	2180	308
DF%	98.4	100	100	90.3	61.3	100	62.9								
Boys (*n* = 132)															
GM	1.76	160	25.4	200	3.66	26.8	11.6		2.37	214	34.2	270	4.94	36.1	15.6
AM	2.52	449	192	492	29.7	70.2	50.3		1.87	888	507	1380	36.2	166	241
Max	24.9	2300	4250	2580	346	346	336		74.3	12,500	9680	22,200	274	1200	5770
DF%	100	100	97.0	99.2	84.4	97.0	43.9								

^a^ GM: geometric mean, to obtain GM, a value of $LOQ/\sqrt{2}$ was assigned to sample with values <LOQ; ^b^ AM: arithmetic mean; ^c^ GM ± GSD (geometric standard deviation) of urinary creatinine was measured as 454 ± 4.01 μg/mL.

High detection frequencies (>90%) were observed for urinary enterolactone, enterodiol, daidzein, and genistein. *O*-DMA (in 184 specimens) and equol (in 136 specimens) were also detected in the children’s urine (Table 1). Among the six target phytoestrogens, enterolactone and daidzein are the predominant compounds, with GM concentrations of 162 and 142 ng/mL, respectively. 

For the urinary enterodiol and genistein, GM concentrations of about 20 ng/mL was detected. Concentration levels of equol and *O*-DMA was lower, with GM values of 10.8 and 4.87 ng/mL, respectively. A significant difference was observed for urinary daidzein in Chinese boys and girls. GM concentration of 200 ng/mL was obtained for urinary daidzein in boys, which is two times that in girls (GM: 101 ng/mL).

## 4. Discussion

**Figure 1:**
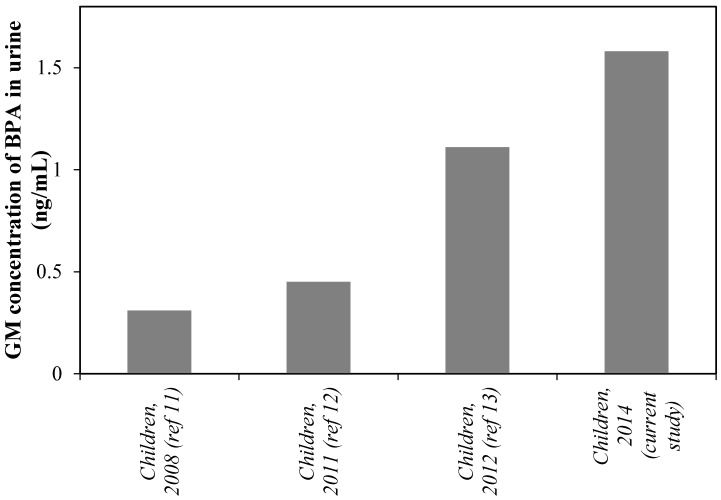
Chronological changes of GM concentration urinary BPA in children from China; the data comes from references [11,12,13] and the results of the current study.

China is one of the biggest consumers of BPA, with an estimated annual BPA demand of about 3 × 10^6^ tonnes [21]. Biomonitoring studies of BPA in the Chinese population have been reported in recent years [11,12,13,14]. For example, He *et al.* reported a wide occurrence of BPA in human urine collected in 2008 from Shanghai, one of the biggest cities in Eastern China, with GM BPA concentrations of 0.31 ng/mL in children aged 8–11 years [11]. Another investigation in 2012 also indicated a GM concentration of 0.45 ng/mL in school children from Shanghai [12]. More recently, Wang *et al.* reported a GM concentration of 1.11 ng/mL for urinary BPA in children from Shanghai [13]. However, the existing studies mainly focus on the population in Eastern and Southern China, and little data is available for estimating the exposure to BPA of people living in Northern China. In the current study, the GM concentration of urinary BPA was measured to be 1.55 ng/mL in children from Tianjin in Northern China. Compared with the previous Chinese children data, an increasing trend of BPA exposure in Chinese children might exist (Figure 1), when ignoring the regional differences between Shanghai and Tianjin. However, all the available data come from some small size biomonitoring studies [11,12,13] and a continuous systematic survey is still required to confirm the BPA exposure trend. 

Concentrations of urinary BPA have been reported by the U.S. Centers for Disease Control and Prevention (USCDC) in their Fourth National Report on Human Exposure to Environmental Chemical [1]. In the newest updated table, BPA was reported to be detected in almost all urine samples taken the U.S., with a GM concentration of 1.51 ng/mL (in the 2011–2012 survey) [1]. This suggests that the BPA exposure of the Chinese children in this study might be at a similar level as that present in U.S. children. 

Two typical isoflavone metabolites, daidzein and genistein, were widely detected in urine from Chinese children in this study. In addition, metabolites of daidzein, *i.e.*, *O*-DMA and equol, were also detected in a part of the urine specimens. Knowledge about urinary phytoestrogen levels in Chinese is limited [22,23,24]. In Chinese pregnant women, GM concentrations of urinary daidzein, enterolactone and enterodiol were detected to be 115, 56.2, and 24.1 μg/g-creatinine, respectively [22]. In addition, GM concentrations of urinary phytoestrogens, *i.e.*, 46.6 μg/g-creatinine for daidzein, 36.6 μg/g-creatinine for genistein, and 5.55 μg/g-creatinine for equol, were reported in Chinese men [23]. These results suggest significantly lower urinary levels of phytoestrogens in Chinese adults than in children. Similar differences between children and adults can also be observed in the U.S. [1]. Besides, a significant gender difference was observed only for urinary daidzein (*p* < 0.01).

**Figure 2:**
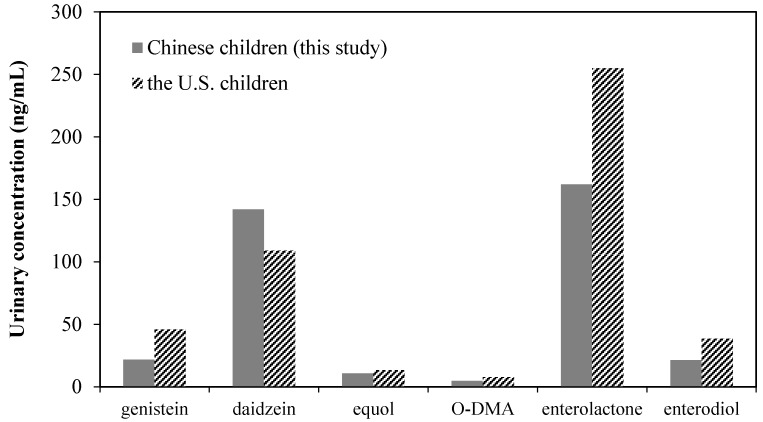
GM concentration of urinary phytoestrogens in children from China and the U.S. U.S. data from reference [1].

According to the data from the U.S. National Health and Nutrition Examination Survey [1], the urinary concentrations of enterolactone (GM: 255 ng/mL) and genistein (GM: 46.1 ng/mL) in 6–11 years children from the U.S. were significantly higher than those detected in Chinese children (*p* < 0.05). On the contrary, concentration of urinary daidzein in the U.S. children (GM: 109 ng/mL) is lower than that in Chinese children (Figure 2).

Significant correlations were found between the homologous phytoestrogens, e.g., enterolactone and enterodiol (*R* = 0.530, *p* = 0.000), daidzein and genistein (*R* = 0.744, *p* = 0.000), daidzein and equol (*R* = 0.608, *p* = 0.000), (Table 2). However, no obvious correlation was found between BPA and phytoestrogens.

**Table 2:** Results of Spearman’s correlation analysis between analytes.

	BPA		Enterodiol		Daidzein		*O*-DMA		Genistein		Equol	
	*R*	*p*	*R*	*p*	*R*	*p*	*R*	*p*	*R*	*p*	*R*	*p*
enterolactone	0.224	0.088	0.530	0.000	0.097	0.481	0.230	0.142	−0.010	0.940	0.462	0.008
enterodiol	0.073	0.583			0.324	0.016	0.326	0.033	0.251	0.057	0.142	0.448
daidzein	0.301	0.024					0.338	0.031	0.744	0.000	0.608	0.000
*O*-DMA	−0.037	0.815							0.095	0.544	0.222	0.376
genistein	0.193	0.143									0.321	0.079
equol	0.407	0.021										

## 5. Conclusions

In this study BPA was widely detected in urine from children in Tianjin, China, at a concentration level higher than that reported for Chinese children in the past. The relatively high urinary BPA concentration level in this study indicates an increasing risk of BPA exposure to Chinese children. In addition, attention should be paid to the human exposure to BPA substitutes, such as bisphenol S (BPS), bisphenol F (BPF), and bisphenol B (BPB). While intake of phytoestrogens has been frequently associated with health benefits, potentially adverse effects on development, reproductive and endocrine systems are being observed [25]. Levels of urinary phytoestrogens in Chinese children are lower than the reported levels in Chinese adults. In addition, compared with the concentration levels in the U.S. children, significantly higher urinary daidzein levels were observed in the Chinese children, especially in Chinese boys, that should be of concern. 
